# A Chinese alligator in heliox: formant frequencies in a crocodilian

**DOI:** 10.1242/jeb.119552

**Published:** 2015-08

**Authors:** Stephan A. Reber, Takeshi Nishimura, Judith Janisch, Mark Robertson, W. Tecumseh Fitch

**Affiliations:** 1 Department of Cognitive Biology, University of Vienna, Vienna 1150, Austria; 2 Primate Research Institute, Kyoto University, Inuyama, Aichi 484-8506, Japan; 3 St Augustine Alligator Farm Zoological Park, St Augustine, FL 32080, USA

**Keywords:** Bioacoustics, Source-filter theory, *Alligator sinensis*, Vocal tract resonance, Bellow, Archosauria

## Abstract

Crocodilians are among the most vocal non-avian reptiles. Adults of both sexes produce loud vocalizations known as ‘bellows’ year round, with the highest rate during the mating season. Although the specific function of these vocalizations remains unclear, they may advertise the caller's body size, because relative size differences strongly affect courtship and territorial behaviour in crocodilians. In mammals and birds, a common mechanism for producing honest acoustic signals of body size is via formant frequencies (vocal tract resonances). To our knowledge, formants have to date never been documented in any non-avian reptile, and formants do not seem to play a role in the vocalizations of anurans. We tested for formants in crocodilian vocalizations by using playbacks to induce a female Chinese alligator (*Alligator sinensis*) to bellow in an airtight chamber. During vocalizations, the animal inhaled either normal air or a helium/oxygen mixture (heliox) in which the velocity of sound is increased. Although heliox allows normal respiration, it alters the formant distribution of the sound spectrum. An acoustic analysis of the calls showed that the source signal components remained constant under both conditions, but an upward shift of high-energy frequency bands was observed in heliox. We conclude that these frequency bands represent formants. We suggest that crocodilian vocalizations could thus provide an acoustic indication of body size via formants. Because birds and crocodilians share a common ancestor with all dinosaurs, a better understanding of their vocal production systems may also provide insight into the communication of extinct Archosaurians.

## Abstract (precis)

**Highlighted Article:** Frequency peaks in bellows of a Chinese alligator breathing a helium–oxygen mixture instead of air shift to significantly higher frequencies, showing that crocodilian vocalizations contain vocal resonance frequencies or ‘formants’.

## INTRODUCTION

In many vertebrates, long-distance vocalizations convey cues to a caller's physical attributes, such as body size (Hardouin et al., 2007, 2014; Harris et al., 2006; Vannoni and McElligott, 2008). In mammals, formant frequencies (vocal tract resonances) represent a widespread mechanism for producing such honest acoustic signals (Charlton et al., 2012, 2011; Fitch and Hauser, 2002; Reby et al., 2005). Formants are often negatively correlated with a caller's vocal tract length and hence its size (Fitch, 1997; Reby and McComb, 2003; Riede and Fitch, 1999). Bird vocalizations possess formants (Nowicki, 1987) and although they have several functions, it is thought that in at least some species they can serve (Fitch, 1999; Hinds and Calder, 1971; Jones and Witt, 2014; Miller et al., 2007) and be perceived as honest signals of body size (Budka and Osiejuk, 2013; Fitch and Kelley, 2000). However, there is little evidence for formants in anurans (Rand and Dudley, 1993), and to date formants have not been shown to play a role in the vocalizations of non-avian reptiles.

Crocodilians are among the most vocal non-avian reptiles (Britton, 2001; Campbell, 1973), and adults of either sex produce loud vocalizations, called ‘bellows', year round with the highest occurrence during the mating season (Vergne et al., 2009). It has been suggested that bellows serve as advertisement calls, carrying cues to sex and size (Garrick and Lang, 1977). In all extant crocodilian species, relative size differences strongly affect both courtship and agonistic behaviour. Females only accept males larger than themselves as mates (Brazaitis and Watanabe, 2011), and size reliably predicts the outcome of agonistic interactions (Tucker et al., 1998; Webb et al., 1983). Accurate acoustic cues to body size would facilitate several aspect of crocodilians' social behaviour, because both sexes compete over breeding grounds and mates (Garrick and Lang, 1977). In some crocodilian species, bellow displays differ between sexes; for example, in American alligators (*Alligator mississippiensis*), only males appear to exhibit sub-audible vibrations (visible by the ‘water dance’) before the audible bellow (Vliet, 1989). Additionally, bellowing seems to stimulate equivalent vocal responses in other conspecifics and to attract individuals towards the sound source (Wang et al., 2009b). Finally, bellows are rather noisy vocalizations with a broad energy spectrum (Garrick and Lang, 1977), which would make these calls well suited to indicate size with formants.

No study to date has demonstrated that crocodilian bellows exhibit formants; indeed, the existence of formants has not been demonstrated in any non-avian reptile. A powerful method to identify the presence of formants is to compare an animal's vocalizations produced in the atmosphere conditions of ambient air and heliox, a helium/oxygen mixture (Herrsch, 1966; Madsen et al., 2012; Nowicki, 1987; Rand and Dudley, 1993; Roberts, 1975). Because helium is lighter than air, the speed of sound is higher in heliox, shifting resonances in a gas-filled cavity upward in frequency (Koda et al., 2012). Although heliox allows normal respiration, it sharply alters the distribution of formants in the sound spectrum (Beil, 1962). In contrast, frequency bands produced by the sound source (e.g. vocal fold or other tissue vibrations) will remain unchanged in the two conditions. The heliox method can thus clearly disentangle the independent contributions of the source and the filter to the acoustic output (Fant, 1960; Holywell and Harvey, 1964). The two are currently not known to be coupled in vertebrate vocalizations. By means of the heliox method it was shown, for example, that the vocal sac of four species of anurans does not act as a cavity resonator (Rand and Dudley, 1993) and that source characteristics do not shift frequency appreciably in heliox in humans (Beil, 1962), gibbons (Koda et al., 2012), bats (Hartley and Suthers, 1988; Roberts, 1973) or birds (Brittan-Powell et al., 1997; Gaunt et al., 1987; Nowicki, 1987).

In this study, we sought evidence for formants in crocodilians by comparing bellows produced in air and in heliox by a female Chinese alligator (*Alligator sinensis* Fauvel 1879). As relatively small but highly vocal crocodilians, Chinese alligators are well suited for such experiments (Garrick, 1975; Wang et al., 2009a, 2007). We induced our subject (total length 125 cm) to bellow in an airproof chamber using playbacks of its own vocalizations. The alligator breathed either ambient air or heliox (88% helium, 12% oxygen). We calculated the speed of sound for these two gas mixtures and estimated the subject's vocal tract length using the externally measured head length (16 cm). Based on these values, we performed an automated acoustic analysis of the recordings. Our aims were (1) to identify a source signal, which should remain unchanged in the two conditions (Taylor and Reby, 2010), and (2) to determine whether the calls contained formant frequencies, which should be significantly altered in the two different atmospheres.

## RESULTS

Playbacks were highly effective in inducing bellowing in our subject: typically a bout of two call playbacks elicited a bellowing response. The animal bellowed in every section of the experiment on two testing days. For all analysis steps, the recordings from these 2 days were pooled and compared by atmosphere treatment.

### Source signal, intensity and duration

Bellows of Chinese alligators are relatively chaotic vocalizations with very little harmonic signal, and the fundamental frequency could not be measured with confidence. However, the bellows do typically show a pulsed structure around 35 Hz, which was visible in the spectrogram. We automatically extracted the temporal intervals between these pulses using the Praat acoustic analysis program (version 5.2.45, www.praat.org). Calls in which Praat could not identify the targeted pulses were excluded from the analysis (causing a reduction in sample size). The mean period (in s) between the pulses in a call was used to calculate the pulse frequency. This measurable component of the source signal did not change between bellows produced in ambient air or heliox (exact Wilcoxon rank-sum test, *N*_air_=43, *N*_heliox_=47, *Z*=−0.064, *P*=0.949). We also extracted the frequency with the highest amplitude in the spectrum, the dominant frequency, from each recording as an additional potential indicator of the source component of the acoustic output. The dominant frequency remained essentially constant in all bellows recorded, and the values did not differ significantly between the two conditions (*N*_air_=58, *N*_heliox_=68, *Z*=−0.405, *P*=0.686). No differences were found between bellows in air and heliox for relative intensity (*Z*=−1.072, *P*=0.284) or call duration (*Z*=−0.818, *P*=0.414; Table 1).

**Table 1.**
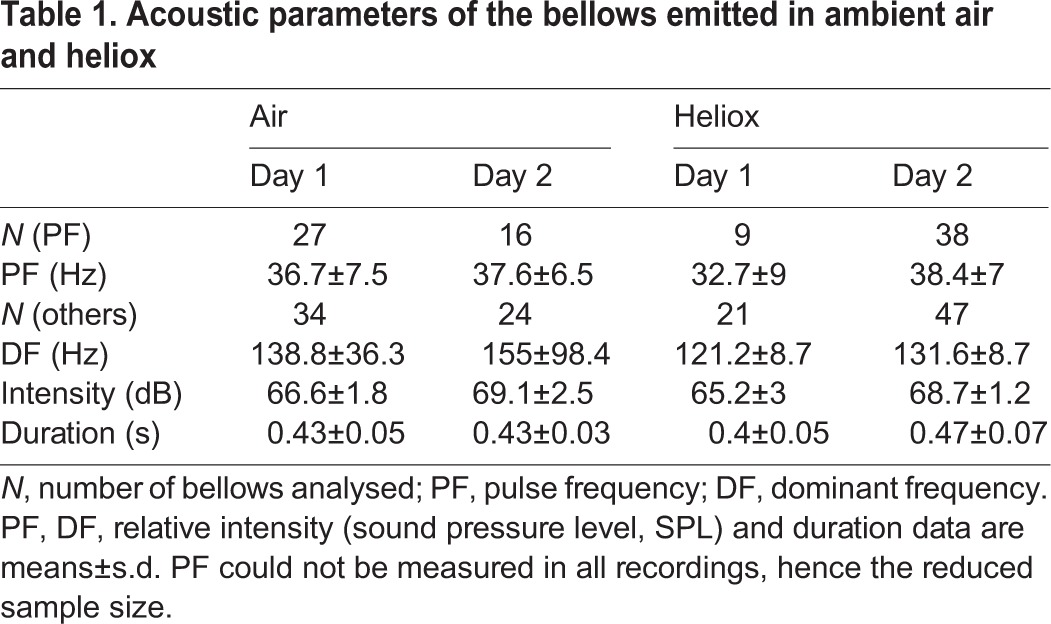
**Acoustic parameters of the bellows emitted in ambient air and heliox**

### Formant frequencies

In order to automatically measure the first two formants, the Praat settings were adjusted to roughly match the expected location of the resonance frequencies in the spectrum based on the subject's estimated vocal tract length (16 cm), the outside/body temperature (∼23°C) and the chemical composition of the two atmospheres [Praat command: To Formant (burg); Maximum number of formants: 2; Window length: 0.05 s; Maximum formant: 2 kHz air, 4 kHz heliox; Pre-emphasis from: 400 Hz air, 800 Hz heliox]. Recordings in which Praat could not identify two formants were not considered in the analysis (causing a reduction in sample size). There was a highly consistent shift of high-energy frequency bands between the two atmospheres. The frequencies identified by the software as the first formant (exact Wilcoxon rank-sum test, *N*_air_=49, *N*_heliox_=52, *Z*=−3.106, *P*=0.002) and the second formant (*Z*=−11.32, *P*<0.001) were consistently and significantly higher in the heliox than in the normal air condition (Table 2).

**Table 2.**
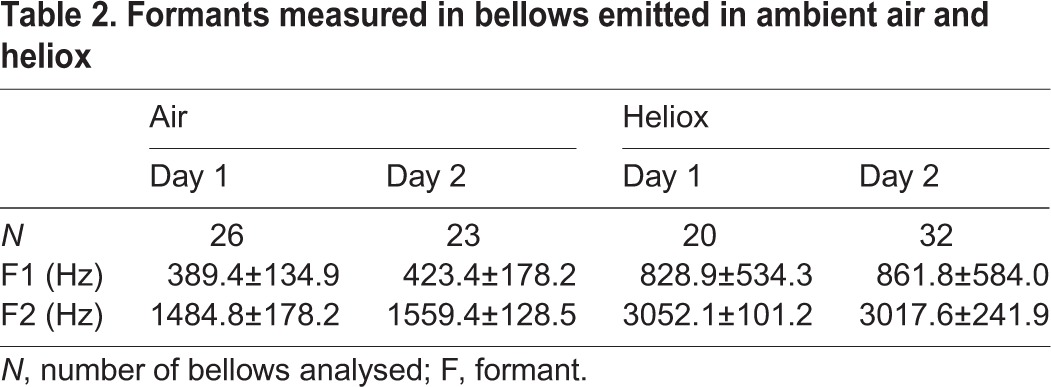
**Formants measured in bellows emitted in ambient air and heliox**

**Fig. 1.**
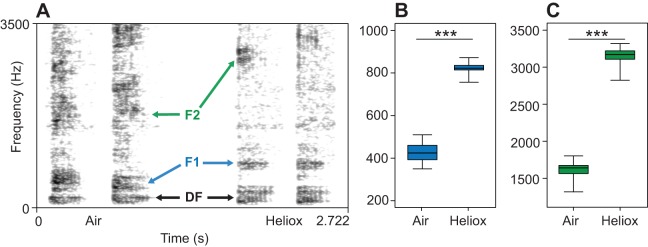
**Formants of bellows produced in heliox are higher than in ambient air.** (A) The spectrogram shows two calls in air (left) and two calls in heliox (right). DF, dominant frequency; F, formant (Praat settings: Method, Fourier; Window shape, Gaussian; Window length, 0.05 s; Dynamic range, 35.0 dB). (B,C) Frequencies of the first formant (B) and second formant (C) in the two conditions; boxplots represent the 25th and 75th percentiles, the centre line indicates the median, whiskers indicate the full data range (***P<0.001).

Next, a subsample of call recordings with a high signal-to-noise ratio was selected from both treatments for a more detailed analysis. The first two formants were measured (Query: Formant listing) by manually adjusting the settings to create a good visual overlap between the formant frequencies as indicated in Praat's editing window and the frequency bands of high energy visible in the spectrogram. The first formant was identified on average at 425.7±46.0 Hz (mean±s.d.) in air and at 825.1±26.1 Hz in heliox. For the second formant, the average frequencies were at 1618.4±112.7 Hz in air and at 3155.4±119.5 Hz in heliox. Both formants were significantly higher in heliox than in air (exact Wilcoxon rank-sum test, *N*_air_=16, *N*_heliox_=16, *Z*=−5.915, *P*<0.001; Fig. 1), as in the automatically measured recordings (see supplementary material Audio 1).

## DISCUSSION

High-energy frequency bands in the bellows of the Chinese alligator were shifted towards higher frequencies when the animal vocalized in the heliox condition. Because changing the gas mixture affects the resonances of the gas-filled vocal cavities while leaving the properties of the vibrating tissues unchanged, we conclude that these shifted high-frequency energy bands represent formant frequencies created by resonances in the vocal tract, rather than components of the sound source. To our knowledge, this is the first clear evidence for formants in non-avian reptiles.

**Fig. 2.**
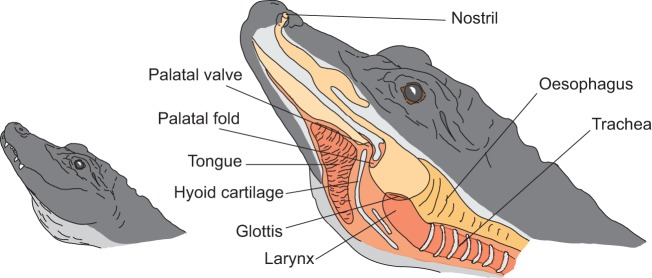
**Proposed vocal tract configuration of a Chinese alligator during bellowing.** Based on photographs, videos and observations, the drawing depicts the proposed arrangement of the supralaryngal vocal tract in the ‘head oblique tail arched posture’ during bellowing; terminology is derived from Britton, 2001 and Reese, 1945; further based on dissections and CT scans of American alligators by W.T.F. and S.R.

The pulse frequency and the dominant frequency were both unaffected by the change in atmosphere composition. While we expected the pulses to represent a component of the source, the dominant frequency could also have been linked to filter properties. Our results show that both components are indicators of the source signal, while neither of them necessarily represents the vocal fold vibration rate. Although vocal fold vibrations might have been the origin of these two constant components, there are also other vocal structures that could vibrate in the air stream, for example the tissue on the outer rim of the palatal valve or the palatal fold (Fig. 2). In the spectrogram, the first formant and the dominant frequency formed a broad frequency band in the ambient air condition. A previous study on Chinese alligator bellows predicted that the source signal was to be found at the lower half of this broad frequency band (Garrick, 1975), and our study provides clear support for this hypothesis. Studying vocal tract resonances with the heliox approach allowed a clear and objective discrimination between the contributions of the source and the filter. Thus, crocodilians should in principle be able to convey cues to their body size in their bellows via formant frequencies. Further research is necessary to test this prediction.

It remains unknown precisely which supralaryngal structures contribute to the vocal tract resonances in crocodilians. All crocodilian species possess a palatal valve (Britton, 2001) consisting of the hyoid cartilage covered by epithelium and connective tissue (Reese, 1945). This structure can be lifted up and pressed against the so-called palatal fold, analogous to the mammalian epiglottis, which prevents water from flowing into the glottis. This sealable space behind the palatal valve delimits the pharynx and the highly expandable crop of the oesophagus. A pair of internal choanae connect this pharyngeal area to the nostrils via the nasal cavity. Juvenile crocodilians can produce their tonal distress calls with either an open or a closed palatal valve (Britton, 2001; Herzog and Burghardt, 1977), so that either the nasal cavity or the shorter oral passage between the glottis and the oral opening could constitute the vocal tract in such calls.

If bellows serve as advertisement calls, with formants as cues to size, crocodilians might be expected to extend their vocal tracts to maximum length during bellows, as seen in several deer species (Fitch and Reby, 2001; McElligott et al., 2006). Shortly before a bellow, the animal elevates its head and lifts its tail out of the water (‘head oblique tail arched posture’; Garrick et al., 1978). In this position an expanded pouch appears below the larynx area, which potentially contains the massive tongue. It is possible that the tongue is thus pulled towards the sternum by intrinsic muscles, thereby pressing the palatal valve firmly against the palatal fold and moving the larynx further caudally. With the oral opening sealed and the pharyngeal cavity extended, the vocal tract (also including the nasal cavity) would reach its maximum length (Fig. 2). X-rays of vocalizing animals would be necessary to test this hypothesis.

From a phylogenetic viewpoint, our study extends the source-filter theory (Fant, 1960; Fitch and Hauser, 2002; Taylor and Reby, 2010; Titze, 1994) to another taxonomic group, making it likely that formants are important for acoustic communication not just in mammals and birds but in amniotes in general. Future research should determine whether formants negatively correlate with body size, and playback experiments would clarify whether crocodilians perceive these cues and behave appropriately. If bellows are advertisement calls as has often been suggested (Brazaitis and Watanabe, 2011), this aspect of crocodilian vocal communication could develop into an intriguing area of study in vertebrate behavioural ecology/comparative cognition. In red deer, females perceive cues to size in male vocalizations and show a preference for larger males (Charlton et al., 2007). In crocodilians, however, both sexes vocalize and actively court (Garrick et al., 1978), and the relative size of potential mates or competitors could be a deciding factor in the decision whether to approach a bellowing conspecific.

The overall morphology of modern crocodilians is highly conserved across species, and all species produce bellow-like vocalizations, suggesting that all crocodilians may possess formants. Birds and crocodilians are the only extant Archosaurians and share a common ancestor with all extinct dinosaurs. It has been suggested that resonance frequencies played a biological role in dinosaur vocalizations (Weishampel, 1981), and the present findings demonstrating formants in crocodilians, combined with previous evidence for avian formants cited above, provide new empirical support for this intriguing hypothesis.

## MATERIALS AND METHODS

### Subject

Data were collected at the St Augustine Alligator Farm Zoological Park, FL, USA, in June 2013. An adult Chinese alligator (*A. sinensis*) female (head length 16 cm, snout–vent length 64.5 cm, total length 125 cm) had undergone and recovered from medical treatment and was quarantined in a rectangular plastic tub (L×W×H=120×110×81 cm) for observation. It bellowed frequently, usually in response to the bellowing chorus of 40 American alligators (*A. mississippiensis*) in a nearby enclosure.

### Call recordings and bellowing stimulation

The alligator's vocalizations were recorded with a Sennheiser directional microphone (ME 66) connected to a digital sound recorder (Zoom H4n Handy Mobile 4-Track Recorder, at 44.1 kHz sampling frequency and 16-bit amplitude resolution). The microphone was mounted on a tripod and its tip was placed at a constant distance of 100 cm from the closest corner. A battery-powered loudspeaker (Ion Audio IPA16 Block Rocker AM/FM Portable PA System, frequency response: 70–20,000 Hz) was placed onto an oblong wooden crate, one edge of which rested on the rack supporting the quarantine tub. The loudspeaker was placed 50 cm away from the tub. The alligator could be reliably induced to bellow by playing back a sound file with the subject's own calls arranged into bouts of three calls. When playing sounds, the loudspeaker transmitted vibrations both via air and into the water, and thus to the animal inside the tub.

### Construction and training prior to the experiment

The sidewalls of the quarantine tub were made airproof by filling holes and gaps with Premium waterproof silicone. Using polyvinyl chloride (PVC) pipes and plastic seal washers, a gas inlet and a sealable vent were built into two opposing walls. Two gas cylinders filled with pure oxygen and helium, respectively, were connected via plastic tubes to the gas inlet. To accurately monitor the water level and pressure changes inside the opaque tub, a column manometer was installed on the outside of the front wall. The tub had a removable lid with a central window covered with wire mesh that allowed visual access to the subject. The area of the lid that was in contact with the rim of the tub was coated with a 1 cm-high bead of silicon. In order to make the entire system airtight, a plastic drop cloth was laid over the top opening of the tub, the lid was lowered onto it, and four flagstones were placed on each corner, which compressed the silicon bead and sealed the tub. For several days prior to the experiment, the water level inside the tub was repeatedly raised and lowered for two purposes: to train the alligator for the upcoming experimental procedure and to determine the maximum water level at which it could still comfortably bellow. During disturbances, floating crocodilians typically submerge defensively, and our alligator submerged immediately when any water flow occurred.

### Experimental procedure

**Fig. 3.**
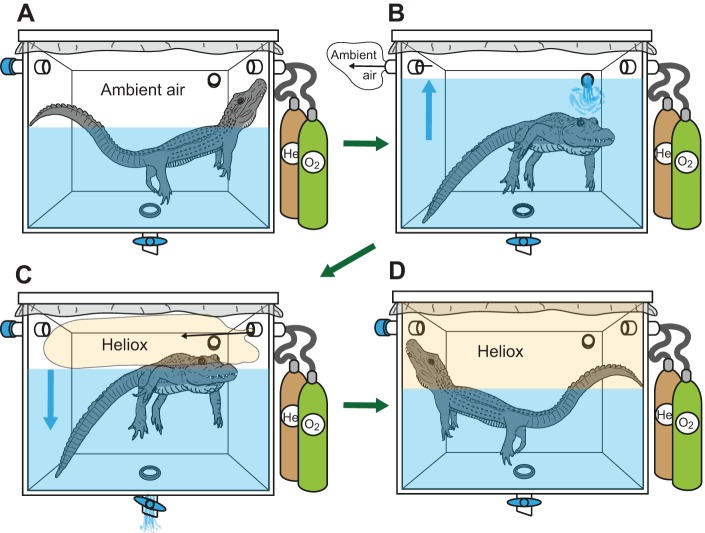
**Atmosphere exchange during the experimental procedure without handling the subject.** After inducing the alligator to bellow in the sealed chamber in ambient air (A), the water level was raised and the ambient air was removed (B). While lowering the water level again, the vacuum was filled with heliox (C) and the animal was subsequently stimulated to bellow in the heliox atmosphere (D).

The entire experiment was performed without handling the animal (Fig. 3). The lid was put on the tub and the setup sealed. Then the air vent was opened, the water level was raised to maximum bellowing height (63 cm of water, 18 cm of air), and the vent was sealed again. The alligator was induced to bellow in air for 5 min (Fig. 3A). Next, the air vent was opened again and the tub filled with water to the very top (Fig. 3B). After sealing the air vent, the water level was slowly lowered and the resulting vacuum was filled with the helium–oxygen mixture until the maximum bellowing height was reached (Fig. 3C). The helium was piped in first, followed by the oxygen. By monitoring the column manometer, a heliox mixture of 88% helium and 12% oxygen was created. After a 5 min break to allow full replacement of the alligator's respiratory gases by heliox, the alligator was again stimulated to vocalize (Fig. 3D) using playbacks (44–46 min). Afterwards, the water level was again raised to the top to eject the heliox. After opening the air vent, the water was lowered to the maximum bellowing height, so that ordinary atmospheric air once again filled the space above the water surface. The tub was again made airtight and the alligator was stimulated with the playback for a third time (22–24 min). This entire procedure was repeated again 3 days later, yielding a total of four atmospheric and two heliox bellowing bouts.

### Acoustic analyses

Individual bellows were extracted from the recordings using Adobe Audition (version 4.0), and recorded calls were discarded if they overlapped with the stimulation playback calls. All acoustic analyses were conducted using the Praat analysis program (version 5.2.45, www.praat.org). Our analysis focused on the following three steps: (1) source signal, intensity and duration, measured automatically; and formant frequencies measured (2) automatically and (3) manually, as detailed below.

#### Source signal, intensity and duration, measured automatically

The pulse frequency was calculated from the mean pulse period, which was automatically measured by the software. By adjusting the pitch settings [To PointProcess (periodic, cc); Minimum pitch: 20 Hz; Maximum pitch: 70 Hz] a visual match was created between the pulse indicators (Show pulses) in the waveform and the visible pulses in the time domain of the spectrogram. Subsequently, the mean pulse period was obtained from the PointProcess object [Get mean period; Time range: 0.0–0.0 (=all) sec; Shortest period: 0.018 s; Longest period: 0.04 s; Maximum period factor: 1.3]. The dominant frequency, the frequency with the highest amplitude in the spectrum, was also automatically extracted from each recording (To Ltas; Bandwidth: 10 Hz). Additionally, relative intensity and call duration were analysed to clarify whether the two gas treatments had other differential acoustic effects on the alligator's calling behaviour.

#### Formant frequencies, measured automatically

Two higher-frequency peaks in the spectrum were clear in most calls, which we hypothesized to be formants; we used two analysis techniques to measure their frequencies. The putative formants were first automatically measured in all recordings in Praat [To Formant (burg) function]. The analysis settings were adjusted to the physical predictions for the two atmospheres. The temperature at the beginning of the experiments was measured at around 23°C and assumed to be the subject's body temperature, as the experiments were conducted in the morning hours and the tub had had no time to heat up substantially. Hence *c* (speed of sound) was expected to be 345 m s^−1^ in normal air. Given the same temperature and a gas mixture of precisely 88% helium and 12% oxygen, the sound velocity was expected at 712 m s^−1^ in heliox, about twice the velocity in ambient air. The head length of the Chinese alligator was 16 cm, and this measurement was used as a rough approximation of vocal tract length (nasal plus pharyngeal cavity). We approximated the supralaryngal vocal tract as a tube, open at the nostrils and closed at the glottis (Reby and McComb, 2003). With this set of assumptions and following the principles of voice production (Titze, 1994), we calculated the theoretical positions of the first two formants in the spectrum of our recordings from both atmospheres using Eqn 1 (where *c* is the speed of sound and VLT is vocal tract length; Reby and McComb, 2003):
(1)
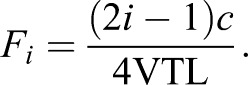


This gives predictions for F1 (first formant) at around 539 Hz and F2 at ∼1618 Hz in ambient air. In heliox, we expected to find F1 at ∼1112 Hz and F2 at ∼3337 Hz. Consequently, the settings for the automated analyses in Praat were adjusted for the two atmospheres [To Formant (burg); Maximum number of formants: 2; Window length: 0.05 s; Maximum formant: 2000 Hz (air), 4000 Hz (heliox); Pre-emphasis from: 400 Hz (air), 800 Hz (heliox)].

#### Formant frequencies, measured manually

We selected 16 call recordings with a high signal-to-noise ratio from each treatment for a more detailed manual analysis of the first two formants. Basic Praat settings were again adjusted to fit the expected sound velocity per atmosphere [Maximum number of formants: 2; Maximum formant frequency: 2000 Hz (air), 4000 Hz (heliox)]. For each recording, additional program settings (Window length, Pre-emphasis) were adjusted to fit the specific call*.* The formants were measured at a point in time (Query: Formant listing) where a visual match was created between the formant frequencies as indicated in Praat's editing window and the frequency bands of high energy visible in the spectrogram.

For all formant analyses, we performed the analyses above with and without downsampling (Resample; New sampling frequency: 10,000 Hz; Precision: 50 samples) and the results were the same; the precise values presented here are without any downsampling.

### Statistical analyses

Statistical comparisons between the calls from the two atmospheres were conducted using the exact Wilcoxon rank-sum test for unpaired data in R (version 3.0.2). All tests were two-tailed with α=0.05.

### Ethical note

Heliox is commonly used in human clinical treatment, because it is easier to inhale than atmospheric air, and in diving. As a noble gas, helium is chemically inert and hence completely non-toxic. The current study was approved by the St Augustine Alligator Farm research committee in April 2013.

## Supplementary Material

Supplementary Material
