# Supplementary material for: A Chinese alligator in heliox: formant frequencies in a crocodilian
Source: J Exp Biol. 2015 Aug;218(15):2442–7. doi: 10.1242/jeb.119552 (PMC4528706; doi:10.1242/jeb.119552)
Supplement: Supplementary Material [file supp_218_15_2442__index.html]

A Chinese alligator in heliox: formant frequencies in a crocodilian — Supplementary Material 

# A Chinese alligator in heliox: formant frequencies in a crocodilian

## JEB119552 Supplementary Material

- Supplementary Material
