# Supplementary material for: A Chinese alligator in heliox: formant frequencies in a crocodilian
Source: J Exp Biol. 2015 Aug;218(15):2442–7. doi: 10.1242/jeb.119552 (PMC4528706; doi:10.1242/jeb.119552)
Supplement: Supplementary Material [file supp_218.15.2442_JEB119552supp.pdf]

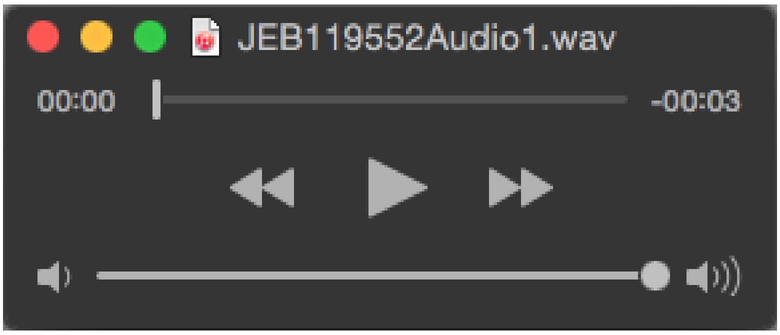

Audio 1. Bellow recordings. The sound file used to create the spectrogram in Fig. 1; two calls in ambient air are followed by two calls in heliox.
